# Impact of neoadjuvant treatment on rectal gastrointestinal stromal tumors

**DOI:** 10.1371/journal.pone.0270887

**Published:** 2022-09-09

**Authors:** Chinock Cheong, Jeonghyun Kang, Byung Soh Min, Nam Kyu Kim, Joong Bae Ahn, Kang Young Lee

**Affiliations:** 1 Department of Surgery, Korea University Guro Hospital, Korea University College of Medicine, Seoul, Korea; 2 Graduate School, Yonsei University College of Medicine, Seoul, Korea; 3 Division of Colorectal Surgery, Department of Surgery, Gangnam Severance Hospital, Yonsei University College of Medicine, Seoul, Korea; 4 Division of Colorectal Surgery, Department of Surgery, Colorectal Cancer Clinic, Severance Hospital, Yonsei University College of Medicine, Seoul, Korea; 5 Division of Colorectal Surgery, Department of Surgery, Yongin Severance Hospital, Yonsei University College of Medicine, Seoul, Korea; 6 Division of Medical Oncology, Department of Internal Medicine, Yonsei Cancer Center, Yonsei University College of Medicine, Seoul, Korea; Tata Memorial Centre, INDIA

## Abstract

Although gastrointestinal stromal tumors (GISTs) are rare disease and rectal GISTs is only 5% of total GISTs, they have the worst prognosis. Due to narrow pelvis, tumor rupture or positive resection margin are common in the management of rectal GISTs. The impact of neoadjuvant treatment on the clinical outcomes of rectal gastrointestinal stromal tumors (GISTs) remains unclear. Thus, we conducted a retrospective study to investigate the impact of neoadjuvant imatinib on rectal GIST. The cohort comprised 33 patients; of them, 10 and 23 belonged to the neoadjuvant (i.e., those who underwent neoadjuvant imatinib treatment) and the control group (i.e., those who underwent surgery without prior imatinib treatment), respectively. Neoadjuvant group was associated with more common levator ani muscle displacement (*P* = 0.002), and showed significantly larger radiologic tumor size (*P* = 0.036) than the control group. The mean tumor size was significantly decreased after imatinib treatment (6.8 cm to 4.7cm, *P* = 0.006). There was no significant difference in resection margin involvement (*P* >0.999), and sphincter preservation rates (*P* = 0.627) between the two groups. No difference was observed with respect to morbidities, hospital stay, local recurrence and disease-free survival. Neoadjuvant imatinib treated group had similar propensity with control group after treatment. We thought reduced tumor sized could enhance resectability and provide more chance to preserve sphincter for rectal GIST patients. Considering large tumor size and higher rate of sphincter invasion in the neoadjuvant group, imatinib treatment could be helpful as a conversion strategy to make huge and low-lying rectal GIST operable and achieve better surgical outcomes.

## Introduction

Gastrointestinal stromal tumors (GISTs) are rare, with an annual incidence of 1/100,000, but they can occur at any site in the gastrointestinal tract [1]. Several prognostic factors, including tumor size, mitotic count, and tumor location, have been used to stratify the risk of recurrence after complete tumor resection [2]. Among GISTs, rectal GISTs have the worst prognosis, with a reported high recurrence rate as local recurrence 87.5%, systemic recurrence 58% after curative resection [3–6]. Complete tumor excision is the most effective treatment modality for curative intent for rectal GISTs. Regional lymph node dissection is not a mandatory procedure because GISTs do not metastasize to regional lymph nodes [2].

Imatinib (imatinib mesylate, Gleevec, Novartis, Basel, Switzerland) has been proven to be effective for the management of metastatic GISTs, including as adjuvant therapy after complete tumor excision [7, 8]. Furthermore, Imatinib showed clinical efficacy in converting unresectable GISTs [9, 10].

For the surgical management of rectal GISTs, several specific factors should be considered. Tumor rupture or unsuccessful excision of tumor are highly aggressive prognostic factors and well-known risk factors for local recurrence in the management of GIST) [11, 12]. Due to the anatomical limitations such as narrow pelvis, tumor rupture or positive resection margin are more common in the perioperative management of rectal GISTs than others. This is translated into higher rate of sphincter-ablation treatment such as abdominoperineal resection. Thus, preservation of sphincter and functional outcome after complete tumor excision is also another considering factor. In this context, the effect of imatinib on downsizing huge rectal GISTs to resectable ones have clinical relevance. Although, various studies reported the conversional impact of imatinib treatments, few studies focused on the role of preoperative imatinib treatment for rectal GISTs with respect to oncologic and functional outcomes. We focused on the conversion strategy of imatinib for rectal GIST. Therefore, this study aimed to investigate the impact of neoadjuvant imatinib treatment in the management of rectal GISTs as a conversion strategy.

## Materials and methods

### Patients and ethical concerns

We evaluated patients who underwent surgical resection and were pathologically diagnosed with rectal GIST located within 15 cm of the anal verge between 2002 and 2014. Data were extracted from a maintained database. Two patients were excluded because of incomplete pathologic results. Finally, 33 patients were included in the analysis. Patients who underwent neoadjuvant imatinib treatment and those who underwent surgery without prior imatinib treatment were classified into the neoadjuvant group and the control group, respectively. This study was approved by the Institutional Review Board of Severance Hospital (IRB No.2019-0682-001) with a waiver of informed consent. This study was also followed to the ethical principles included in Declaration of Helsinki in 1964.

### Patient selection for preoperative imatinib treatment

All patients were preoperatively staged using abdominopelvic computed tomography (APCT) or pelvic magnetic resonance imaging (MRI). Tumor size was defined as the longest distance of the tumor as measured via APCT or pelvic MRI. We repeated APCT or MRI based on the 3–4 months schedule. The surgical treatment was recommended in case the treatment response was anticipated as maximum. Operative methods were decided according to tumor location and size, which were transanal excision with grossly negative margins or radical resection as a low anterior resection or abdominoperineal resection. Complete resection was defined as the removal of the entire tumor, with a negative resection margin. Lymph node resection was not necessary. Although surgical resection was regarded as the treatment of choice, imatinib treatment before resection was selected at the surgeon’s discretion, according to the tumor size or invasiveness to the adjacent organs or anal sphincter by preoperative APCT or MRI. In the neoadjuvant group, preoperative Imatinib was continued until the tumor no longer decreased in size on imaging studies. Surgical resection was planned if the tumor size failed to decrease further or when progression was observed during a regular follow-up.

### Postoperative management and follow-up

Postoperative tumor size and mitotic count were evaluated by pathologists. Risk classification was performed according to the National Institutes of Health consensus criteria (Table 1) [13]. After resection, adjuvant imatinib treatment was used in cases of margin involvement in the pathologic results or GIST recurrence. Recurrence or metastasis was assessed using an APCT scan after surgical resection. Local recurrence (LR) was defined as any pelvic or perineal tumor recurrence, which was radiologically or clinically diagnosed. Systemic recurrence was defined as any recurrence located away from the rectum or adjacent organs in the pelvis. When recurrence was found during follow-up, adjuvant imatinib was administered at 400 mg per day. Dose escalation to 600 or 800 mg per day was applied, according to treatment response. Sunitinib malate (Sutent, Pfizer, New York, USA) was selectively used as second-line chemotherapy.

**Table 1:** The risk classification for gastrointestinal stromal tumor in the National Institutes of health consensus criteria.

Risk category	Tumor size	Mitotic count
Very low	<2cm	<5/50 HPF
Low	2-5cm	<5/50 HPF
Intermediate	<5cm	6-10/50 HPF
	5-10cm	<5/50 HPF
High	>5cm	>5/50 HPF
	>10cm	Any mitotic rate
	Any size	>10/50 HPF

HPF; high-power field

### Statistical analysis

Continuous and categorical variables were analyzed using Student’s *t* test and *x*^*2*^ test, respectively. Fisher’s exact test was used for between-group comparisons. Paired *t-*test was used to compare tumor sizes before and after neoadjuvant treatment. Disease-free survival (DFS) was calculated from the date of first diagnosis to the date of recurrence or the last follow-up date. LR and DFS were assessed using the Kaplan-Meier method. Multivariate analyses for LR and DFS were performed using the Cox proportional hazard model. All statistical analyses were performed using SPSS statistical software (version 20.0, IBM Corp., Armonk, NY, USA). Differences with *P* values of <0.05 were considered statistically significant.

## Results

### Patient characteristics

The cohort comprised 33 patients; of them, 10 and 23 were classified into the neoadjuvant group and the control group, respectively. All ten patients who underwent neoadjuvant imatinib treatment were confirmed to have rectal GIST via transanal biopsy before imatinib treatment.

**Fig 1:**
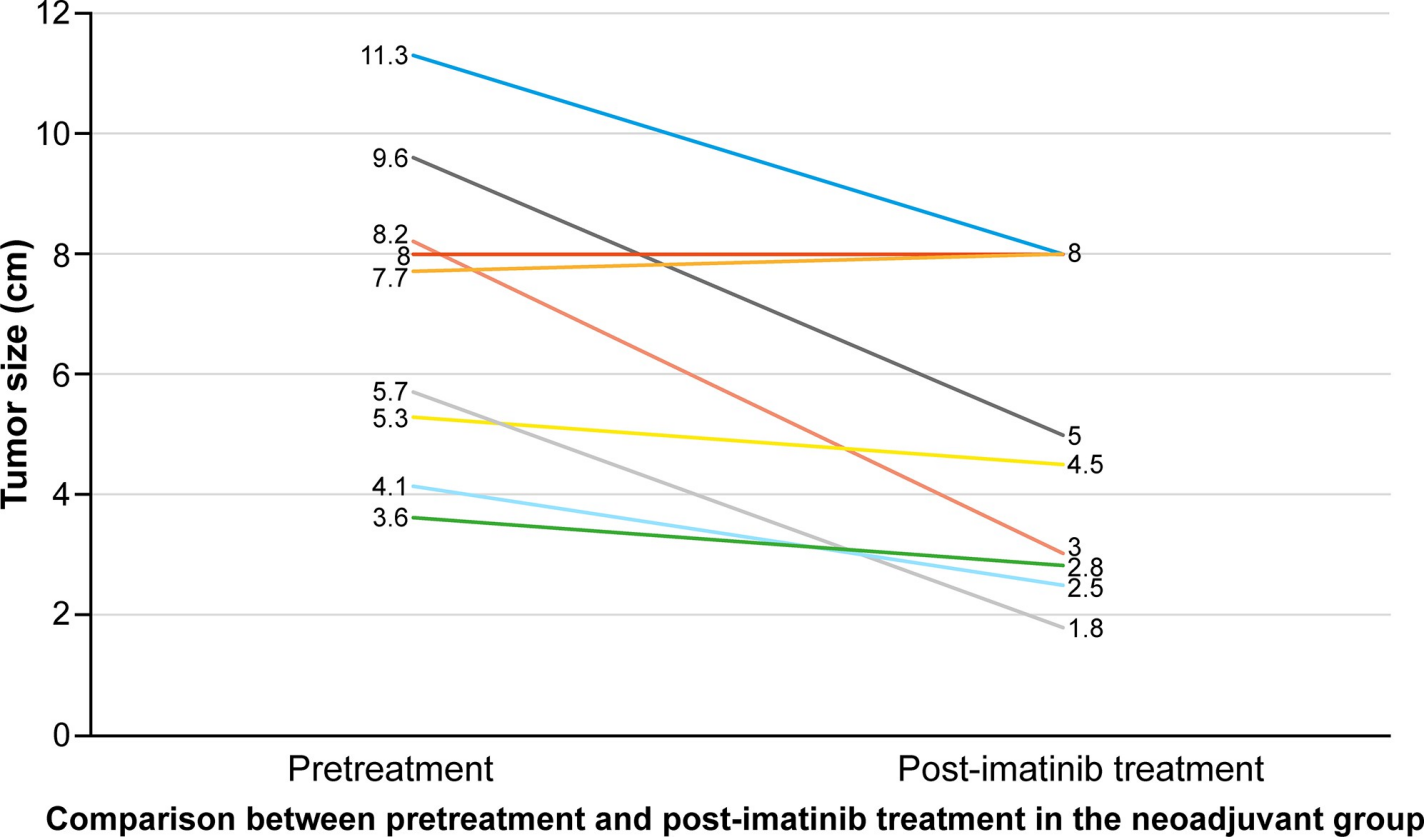
Changes in tumor size after neoadjuvant treatment and comparison of oncologic outcomes between the neoadjuvant and the control groups.

There were no significant differences in age, sex, body mass index, American Society of Anesthesiologists (ASA) grade, or tumor location from the anal verge between the two groups. Most of the tumors (31 (93.9%)) were located less than 5 cm from the anal verge. At the time of diagnosis, the rate of levator ani muscle displacement by the tumor on APCT or pelvic MRI was significantly higher in the neoadjuvant group than that in the control group (9 (90%) vs. 7 (31.8%), *P* = 0.002). The mean tumor size was significantly larger in the neoadjuvant group (6.8 ± 2.5 cm in the neoadjuvant group vs. 4.6 ± 2.6 cm in the control group, *P* = 0.036). After neoadjuvant treatment, the mean tumor size decreased significantly from 6.8 cm to 4.7 cm in the neoadjuvant group (*P* = 0.033) (Fig 1). The mean duration of neoadjuvant treatment with imatinib was 9 months in the neoadjuvant group (9.7±5.0 months) (Table 2).

**Table 2:** Patient characteristics.

	Neoadjuvant group, n (%) (n = 10)	Control group, n (%) (n = 23)	*P* value
Age (years), (mean ± SD)	54.4 ± 15.9	60.8 ± 14.5	0.292
Sex			0.259
Male	7 (70)	10 (43.5)	
Female	3 (30)	13 (56.5)	
BMI (kg/m^2^), (mean ± SD)	24.7 ± 4.5	23.1 ± 3.5	0.285
ASA grade			0.176
<III	10 (100)	16 (69.5)	
≥III	0	7 (30.4)	
Tumor height from the anal verge (cm)			0.085
5.1–10	2 (20)	0	
≤5	8 (80)	23 (100)	
Displacement or abutments to the levator ani muscle			0.002
Yes	9 (90)	7 (31.8)	
No	1 (10)	16 (69.6)	
Preoperative biopsy			0.002
Yes	10 (100)	7 (30.4)	
No	0	16 (69.6)	
Initial tumor size at diagnosis (cm), (mean ± SD)	6.8 ±2.5	4.6 ± 2.6	0.036
Post-treatment tumor size (cm) (mean ± SD)	5.0 ±2.4		
Follow up (months) (mean ± SD)	74.5 ± 39.1	86.7 ± 50.4	0.504

SD, standard deviation; BMI: body mass index; ASA: American Society of Anesthesiologists

### Postoperative outcomes in the neoadjuvant and control groups

The sphincter preservation rates did not differ between the two groups (neoadjuvant vs. control: 8 (80%) vs. 20 (87%), *P* = 0.627). There was also no difference in resection margin involvement between the two groups (neoadjuvant vs. control: 9 (90.0%) vs. 19 (82.6%), *P* >0.999). With respect to difficulty of surgery, there was no difference in the rate of operation time (more than 4 hours) between the two groups. However, intraoperative blood loss >500 ml was more common in the neoadjuvant group than that in the control group (6 (60%) vs. 4 (17.4%), *P* = 0.035). Postoperative complications and hospital length of stay did not differ between the two groups. Most patients showed c-KIT mutations, and there was no difference in mutation frequencies between the two groups (10 (100%) in the neoadjuvant group vs. 22 (95.7%) in the control group, *P* = 0.351). Both groups showed a similar rate of risk stratification (*P* = 0.291) (Table 3).

**Table 3:** Postoperative outcomes of the neoadjuvant and the control groups.

	Neoadjuvant group, n (%) (n = 10)	Control group, n (%) (n = 23)	*P* value
Type of operation			0.786
Transanal excision	1 (10)	6 (26.1)	
Transabdominal excision	0	1 (4.3)	
LAR	7 (70)	12 (52.2)	
APR	2 (20)	3 (13.0)	
Hartmann operation	0	1 (4.3)	
Sphincter preserving surgery			0.627
Yes	8 (80)	20 (87)	
No	2 (20)	3 (13)	
Stoma formation			0.619
No	3 (30%)	11 (47.8%)	
Temporary stoma	5 (50%)	8 (34.8%)	
Permanent stoma	2 (20%)	4 (17.4%)	
Pathologic tumor size (cm ± SD)	5.0 ± 2.4	4.6 ± 2.6	0.713
Mitosis after operation			>0.999
Mitosis ≤ 5/HPF	7 (70)	15 (65.2)	
Mitosis > 5/HPF	3 (30)	8 (34.8)	
Margin involvement			>0.999
Yes	1 (10)	4 (17.4)	
No	9 (90)	19 (82.6)	
Hospitalization (days), (mean ± SD)	7.3 ± 7.9	5.7 ± 3.9	0.253
Clavien-Dindo classification			0.491
I	1 (50)	2 (22.2)	
III	1 (50)	8 (77.8)	
C-kit positive			0.351
Yes	10 (100)	22 (95.7)	
No	0	1 (4.3)	
Exon mutation			0.134
Exon 11 mutation	7 (70)	9 (39.1)	
Exon 13 mutation	1 (10)	1 (4.3)	
Unknown	2 (20)	13 (56.5)	
Risk classification			0.219
Very low	0	5 (21.7)	
Low	5 (50)	4 (17.4)	
Intermediate	2 (20)	6 (26.1)	
High	3 (30)	8 (34.8)	
Recurrence			0.789
No	7 (70)	16 (69.6)	
Local	3 (30)	6 (26.1)	
Systemic	0	1 (4.3)	
Adjuvant treatment	2 (20%)	6 (26.1%)	0.999
Reason for adjuvant treatment			>0.999
Margin involvement	0	4 (17.4%)	
High risk	0	2 (8.7%)	
Unknown	2 (20%)	0	
Duration of adjuvant Treatment (months), (mean ± SD)	26.6±3.5	11.0±8.6	0.056
Imatinib for relapsed tumor	2 (20%)	6 (26.1%)	>0.999
Death			> 0.999
No	9 (90%)	21 (91.3%)	
Yes	1 (10%)	2 (8.7%)	

LAR, low anterior resection; APR, abdominoperineal resection; SD: standard deviation; HPF, high-power field; ASA: American Society of Anesthesiologists

After resection, 20% (n = 2) of patients in the neoadjuvant group and 26.1% (n = 6) of patients in the control group underwent adjuvant treatment with imatinib (*P*>0.999). Although there was no significance between the groups, patients underwent adjuvant treatment because of margin involvement (n = 4) and high risk (n = 2) in control group (*P*>0.999). In the neoadjuvant group, two patients underwent adjuvant treatment based on the physician’s decision, even though they had low risk after surgery (Table 3).

### Oncologic outcomes

**Fig 2:**
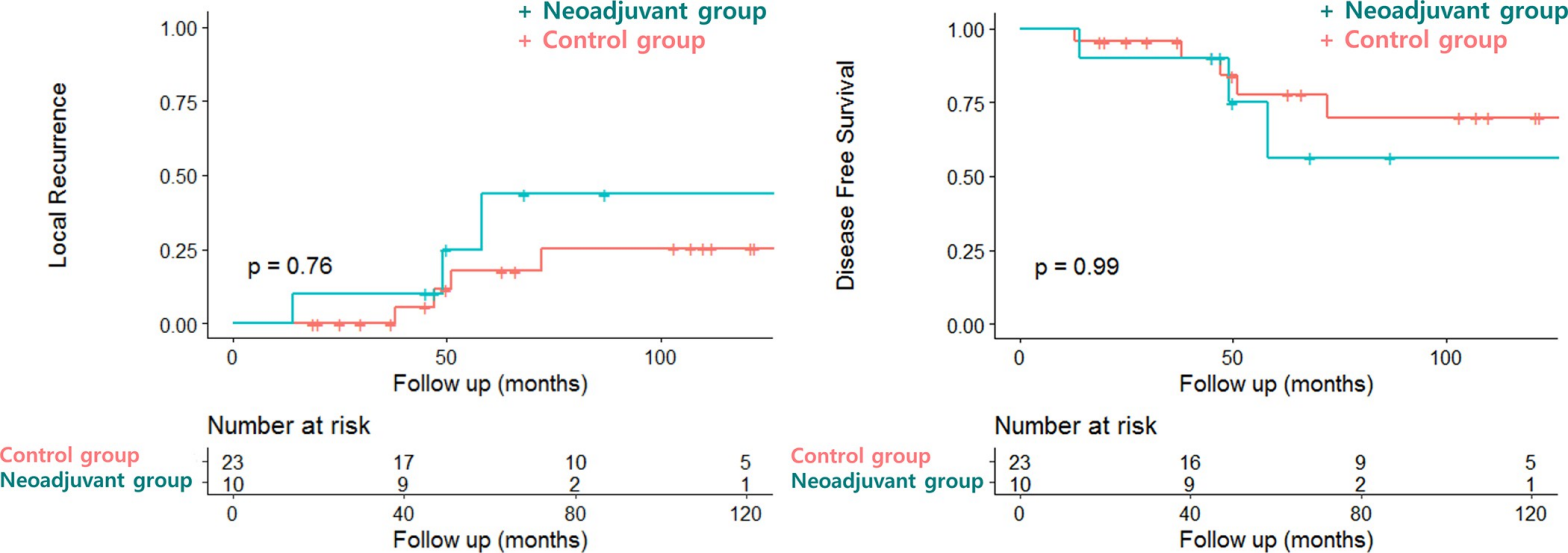
Oncologic outcomes between neoadjuvant and control groups. (a) Local recurrence rates in the neoadjuvant and the control groups. (b) Disease-free survival rates in the neoadjuvant and the control groups.

The 5-year LR rates did not differ between the neoadjuvant and the control groups (neoadjuvant vs. control: 33.3% vs. 17.5%; *P* = 0.76) (Fig 2A). In the multivariate analysis, the prognostic factors of LR were pathologic tumor size (<5 vs. ≥ 5cm) (OR 25.69; 95% CI:2.09–315.32; *P* = 0.011) and exon 11 mutations (OR 10.41; 95% CI: 1.19–91.16; *P* = 0.034). In addition, the 5-year DFS was similar between the groups (neoadjuvant vs. control: 66.7% vs. 77.8%; *P* = 0.99) (Fig 2B). In the multivariate analysis, the prognostic factor of DFS was initial tumor size (<5 vs. ≥ 5cm) (HR 9.501; 95% CI: 1.141–79.13; *P* = 0.037) (Table 4).

**Table 4:** Univariate and multivariate analysis for local recurrence and disease-free survival between neoadjuvant and control groups.

Variables	Local Recurrence				Disease Free Survival			
	Univariate		Multivariate		Univariate		Multivariate	
	OR (95% CI)	P	OR (95% CI)	P	HR (95% CI)	P	HR (95% CI)	P
Sex (male vs. female)	1.48(0.32–6.89)	0.620			0.628(0.152–2.59)	0.52		
Age (<60 vs. ≥60) (year)	1.12(0.24–5.25)	0.886			1.554(0.445–5.422)	0.49		
Sphincter preserving (No vs. Yes)	0.18(0.02–1.35)	0.096			0.443(0.117–1.68)	0.231		
Tumor location (AV <5 vs. ≥5 cm)	2.87(0.165–1.53)	0.473			8.904(0.797–99.55)	0.0759	2.941 (0.254–34.04)	0.388
Initial tumor size (<5 vs. ≥5 cm)	11.20(1.20–104.33)	0.034			9.065(1.124–73.13)	0.0385	9.501 (1.141–79.13)	0.037
Pathologic tumor Size (<5 vs. ≥5 cm)	16.00(1.69–151.11)	0.016	25.69 (2.09–315.32)	0.011	3.776(0.773–18.44)	0.101		
Mitosis (<5 vs. ≥5/HPF)	6.00(1.13–31.73)	0.035			2.756(0.657–11.56)	0.166		
Margin positive	2.00(0.28–14.53)	0.493			1.351(0.279–6.538)	0.709		
Exon 11 mutation	5.83(0.99–34.44)	0.052	10.41 (1.19–91.16)	0.034	4.67(0.987–22.1)	0.052	4.829 (0.983–23.71)	0.052
Neoadjuvant imatinib (No vs. Yes)	1.21(0.24–6.27)	0.817			1.009(0.255–3.993)	0.99		

OR, odds ratio; HR, hazard ratio; CI, confidence interval; AV, anal verge; HPF, high-power field; CTx., chemotherapy

## Discussion

This study demonstrated that neoadjuvant imatinib treatment for large rectal GISTs could reduce tumor size and thus increase the resectability. In addition, it seems not to increase postoperative morbidity and seem not to deteriorate the oncologic outcomes such as resection margin positivity or long term oncologic outcomes.

The National Comprehensive Cancer Network guideline recommends preoperative imatinib for metastatic disease or unresectable cases for which surgery would induce significant morbidity [10]. Nevertheless, there is no consensus on the proper dose or duration of neoadjuvant imatinib especially for the treatment of rectal GISTs. Previous studies have reported the effects of preoperative imatinib for patients with multi-organ or metastatic GISTs [12, 14, 15].

In this study, the duration of neoadjuvant imatinib treatment differed between patients, with a mean duration of 9.7 months. Although changes in tumor density detected on CT scans are known to predict tumor responses after imatinib treatment [16, 17], this prediction modality was not applied to our patients. Maximal responses based on tumor sizes were evaluated by radiologists.

Previously, it has been recommended to use imatinib whenever possible until the effect is insignificant [14, 18, 19]. Nevertheless, it is still unclear how we could decide to stop treatment and proceed to surgery. The adequate duration and dosage of imatinib treatment and imaging parameters that maximize patient response to neoadjuvant imatinib treatment should be evaluated in further studies for rectal GIST.

Another advantage of neoadjuvant treatment is achieving a negative resection margin through a conversion strategy. For rectal GISTs, the rate of positive resection margins is as high as 40% [6], and a positive resection margin is a known independent factor for poor survival [12, 20]. Acquiring a negative resection margin sometimes counters sphincter preservation due to the deep and narrow pelvic cavity. Increasing resectability after neoadjuvant treatment clarifies the resection margin. Cavnar et al. compared neoadjuvant imatinib and control groups of patients who underwent surgical resection for rectal GISTs [21]. Although the ratio of APR in the neoadjuvant group was significantly lower, R1 resection was reported in 30% patients in the neoadjuvant group.

In this study, there was one patient with a positive resection margin in the neoadjuvant group (10%), compared with four patients in the control group (17.4%). We demonstrated the comparison focused on rectal GIST patients and achieved a 90% rate of R0 resection in the neoadjuvant group. The similar resection margin involvement rate and the long-term LR rate indicate that neoadjuvant imatinib treatment can reduce the tumor size and increase its resectability. Therefore, if neoadjuvant imatinib can reduce the size of the tumor and limit its invasion to adjacent organs, it would facilitate complete surgical resection.

In addition to tumor size, mitotic count is also an important risk factor and can determine the malignancy potential. Because mitotic count can be influenced by neoadjuvant imatinib, risk classification might be more accurate when it is evaluated using samples obtained before neoadjuvant treatment. Mutational analysis has recently been found to be essential because it helps exclude less sensitive or resistant tumors to imatinib treatment and allows proper dosing for patients with c-kit exon mutations [22, 23]. Four different regions of kit have been found to be mutated in GIST: exons 9, 11, 13, and 17 [24, 25]. Although most kit mutations are sensitive to imatinib, exon 11 mutations are more sensitive than exon 9 mutations, and exon 17 mutations are resistant to imatinib [26]. Thus, surgical resection should not be delayed in patients who do not show a response. In our analysis, 70% of the patients had exon 11 mutations in the neoadjuvant group, compared with 39.1% in the control group. However, the mitotic count results and exon mutational statuses from preoperative biopsy were not reported. Thus, the impact of imatinib on these parameters could not be analyzed. The selective application of neoadjuvant imatinib treatment according to exon mutation and mitotic count results derived from preoperative biopsy samples is an area of clinical interest and should be evaluated in further studies.

There are several potential limitations in this study. Because of the retrospective study design, we could not ignore selection bias on clinical outcomes. In addition, due to the rarity of rectal GISTs, the number of enrolled patients was relatively small. However, we demonstrated the effect of neoadjuvant imatinib focused on rectal GIST, whereas other studies did not. In our study, the application of neoadjuvant imatinib treatment was left to the surgeon’s decision and/or clinical applicability of imatinib. The use of imatinib in the neoadjuvant setting is not fully reimbursed by the National Health Insurance Corporation in Korea, and there is no standardized indication for performing curative resection. Different follow-up and imaging studies were applied to each patient during imatinib neoadjuvant treatment. Besides there were not enough patients’ functional outcome data or stoma reversal. Because of retrospective study, we could not also evaluate it additionally.

In conclusion, the present study is a comparative analysis of preoperative imatinib treatment between those who underwent neoadjuvant imatinib treatment followed by radical resection and those who underwent surgery without prior imatinib treatment. Our findings showed that neoadjuvant imatinib treatment effected to reduce initial tumor size. Reduced tumor size might increase resectability and thus enhance chance of sphincter preservation for low-lying rectal GISTs. Therefore, neoadjuvant imatinib treatment was worthy of consideration as conversion strategy for huge and low-lying rectal GIST without deterioration long-term outcomes.

## Supporting information

S1 Data (CSV)
